# Pigs’ Feed Fermentation Model with Antimicrobial Lactic Acid Bacteria Strains Combination by Changing Extruded Soya to Biomodified Local Feed Stock

**DOI:** 10.3390/ani10050783

**Published:** 2020-04-30

**Authors:** Laurynas Vadopalas, Modestas Ruzauskas, Vita Lele, Vytaute Starkute, Paulina Zavistanaviciute, Egle Zokaityte, Vadims Bartkevics, Sarunas Badaras, Dovile Klupsaite, Erika Mozuriene, Agila Dauksiene, Sonata Sidlauskiene, Romas Gruzauskas, Elena Bartkiene

**Affiliations:** 1 Lithuanian University of Health Sciences, Mickeviciaus str. 9, LT-44307 Kaunas, Lithuania; laurynas.vadopalas@lsmuni.lt (L.V.); modestas.ruzauskas@lsmuni.lt (M.R.); vita.lele@lsmuni.lt (V.L.); vytaute.starkute@lsmuni.lt (V.S.); paulina.zavistanaviciute@lsmuni.lt (P.Z.); egle.zokaityte@lsmuni.lt (E.Z.); sarunas.badaras@gmail.com (S.B.); dovile.klupsaite@lsmuni.lt (D.K.); erika.mozuriene@lsmuni.lt (E.M.); agila.dauksiene@lsmuni.lt (A.D.); sonata.sidlauskiene@lsmuni.lt (S.S.); 2 Institute of Food Safety, Animal Health and Environment “BIOR”, Lejupes iela 3, Zemgales priekšpilsēta, Riga, LV-1076 Latvia, Lithuania; vadims.bartkevics@bior.gov.lv; 3 Kaunas University of Technology, Radvilenu str. 19, LT-50254 Kaunas, Lithuania; romas.gruzauskas@ktu.lt

**Keywords:** antimicrobial properties, lactic acid bacteria, fermentation, feed, piglets, microbiota, ammonia emission

## Abstract

**Simple Summary:**

The world population is growing, and for this reason, it is very important to ensure increased agricultural production in a sustainable and eco-friendly manner. The aim of this study was to apply a combination of newly isolated antimicrobial characteristic possessing lactic acid bacteria (LAB) strains for local stock (rapeseed meal) fermentation and to evaluate the influence of changing an extruded soya to biomodified rapeseed meal in a feed recipe on piglet feces microbiota, health parameters, growth performance, and ammonia emission. The 36-day experiment was conducted using 25-day-old Large White/Norwegian Landrace (LW/NL) piglets, which were randomly distributed into two groups: a control group fed with a basal diet and a treated group fed with a fermented diet (500 g/kg of total feed). Changing from an extruded soya to fermented rapeseed meal led to desirable changes in piglets’ fecal microbiota (there was more than a four-fold higher *Lactobacillus* count compared to the control group). There was also a 20.6% reduction in ammonia emission in the treated group section. Finally, by changing from extruded soya to less expensive rapeseed meal and applying a fermentation model with selected LAB combination, piglets were fed without any undesirable changes in health and growth performance, as well as in a more sustainable manner.

**Abstract:**

The aim of this study was to apply newly isolated antimicrobial characteristic possessing lactic acid bacteria (LAB) starters (*Lactobacillus plantarum* LUHS122, *Lactobacillus casei* LUHS210, *Lactobacillus farraginis* LUHS206, *Pediococcus acidilactici* LUHS29, *L. plantarum* LUHS135, and *Lactobacillus uvarum* LUHS245) for local stock (rapeseed meal) fermentation and to evaluate the influence of changing from an extruded soya to biomodified local stock in a feed recipe on piglets’ fecal microbiota, health parameters, growth performance, and ammonia emission. In addition, biomodified rapeseed meal characteristics (acidity and microbiological) were analyzed. The 36-day experiment was conducted using 25-day-old Large White/Norwegian Landrace (LW/NL) piglets, which were randomly distributed into two groups: a control group fed with basal diet and a treated group fed with fermented feed (500 g/kg of total feed). The study showed that the selected LAB starter combination can be recommended for rapeseed meal fermentation (viable LAB count in fermented feed 8.5 ± 0.1 log_10_ CFU/g and pH 3.94 ± 0.04). At the beginning of the in vivo experiment, the microbial profiles in both piglet groups were very similar: The highest prevalence was *Prevotella* (34.6–38.2%) and *Lactobacillus* (24.3–29.7%). However, changing from an extruded soya to fermented rapeseed meal in the feed recipe led to desirable changes in piglets’ fecal microbiota. There was a more than four-fold higher *Lactobacillus* count compared to the control group. Furthermore, there was significantly lower ammonia emission (20.6% reduction) in the treated group section. Finally, by changing from an extruded soya to cheaper rapeseed meal and applying the fermentation model with the selected LAB combination, it is possible to feed piglets without any undesirable changes in health and growth performance, as well as in a more sustainable manner.

## 1. Introduction

Considering that the world population is growing, it is crucial to ensure increased agricultural industry production in a more sustainable and eco-friendly manner [1]. According to the prognosis for the near future, the global expectation for livestock products demand will double [2]. Hence, the optimization of animal-based production (reduction of feed prices by using local stock, looking for alternative stock, increasing nutritional value, reduction of greenhouse gas emissions, etc.) is a big challenge for this industry, as well as for scientists. In pig farms, dietary manipulation may positively affect pig growth and reduce production costs. It is also recognized as a possible pollution mitigation strategy [3]. In addition, a prominent challenge comes with bans on the use of antibiotics in animal nutrition. Therefore, all new findings should be utilized to produce feed that meets the nutritional requirements of animals and maintains healthy gut function. For this reason, many antimicrobial-property-possessing compounds have been tested as an alternative to antibiotics to determine whether they improve feed quality. One strategy is feed fermentation. Fermentation leads to many desirable changes, such as preservation of feed, degradation of toxins, and reduction of anti-nutritional factors and non-desirable microorganisms. However, the main challenges for the preparation of fermented feeds are stable and safe usage for longer periods of time [4]. From this point of view, a very important issue becomes the development of new combinations of antimicrobial characteristic possessing starters, which can ensure stable and safe feed fermentation. Our previous studies showed that *Lactobacillus plantarum* LUHS122, *Lactobacillus casei* LUHS210, *Lactobacillus farraginis* LUHS206, *Pediococcus acidilactici* LUHS29, *L. plantarum* LUHS135, and *Lactobacillus uvarum* LUHS245 strains possess antimicrobial properties against various pathogenic and opportunistic strains [5,6]. Antimicrobial properties of lactic acid bacteria (LAB) are crucial because these starters can be used in feed preparation, as an alternative to antibiotics, and they can also promote gut health in pigs. In addition, this strategy may reduce the risk of pathogenic and zoonotic bacteria in the feed-food chain [7]. 

On modern farms, piglets are weaned at an early age, and this step is associated with stress factors, including changes in diet, environment, and social groups. The above-mentioned factors negatively influence feed intake, unbalance the intestinal and immune systems, and lead to an increased risk of primary and secondary infections [8,9]. Finally, the initial growing stages are very important for later production efficiency as they have a marked influence on the pigs’ future health and growth performance [10]. On European pig farms, pharmacological doses of zinc oxide (ZnO) are used to control post-weaning diarrhea; however, starting in 2022 in the European Union (EU), the use of ZnO will be suspended [11]. For this reason, there is an urgent need to find a suitable solution to maintain the performance and gut health of weaner piglets, and the dietary addition of fermented feed has gained attention for its ability to improve production performance and gut health in pig production [12], as well as to enhance the immune system of animals [13,14]. Fermented feed can inhibit intestinal pathogens [15,16,17], improve digestibility, and degrade anti-nutritional compounds in the feed [16,18,19]. Another challenge that should be addressed is reducing greenhouse gas emissions, because agriculture contributes 20–35% to this process [20] and around 80% of total ammonia emission in Europe [21]. In this study, we hypothesized that antimicrobial property possessing LAB can contribute to safe local feed stock incorporation in piglets’ diet, modify the microbiota of piglets, and ensure health and growth performance of the animal. Furthermore, fermented feed is already partly degraded, including degradation of protein to free amino acids [22,23,24]. Therefore, we also hypothesized that fermented feed will reduce ammonia emission.

The aim of this study was to apply newly isolated antimicrobial characteristics possessing LAB starters (*L. plantarum* LUHS122, *L. casei* LUHS210, *L. farraginis* LUHS206, *P. acidilactici* LUHS29, *L. plantarum* LUHS135, and *L. uvarum* LUHS245) for local stock (rapeseed meal) fermentation and to evaluate the influence of changing from an extruded soybean to biomodified local stock in the feed recipe on piglets’ feces microbiota, health parameters, growth performance, and ammonia emission. In addition, biomodified rapeseed meal characteristics (acidity and microbiological) were analyzed.

## 2. Materials and Methods 

### 2.1. LAB Strains Used for Feed Fermentation

The L. plantarum LUHS122, L. casei LUHS210, L. farraginis LUHS206, P. acidilactici LUHS29, L. plantarum LUHS135, and L. uvarum LUHS245 strains were obtained from the Lithuanian University of Health Sciences collection (Kaunas, Lithuania). Our previous studies have shown that the above-mentioned strains inhibit various pathogenic and opportunistic microorganisms and are suitable for various cereal substrates fermentation [5,25,26]. The above-mentioned LAB strains were stored at −80 °C in a Microbank system (Pro-Lab Diagnostics, Merseyside, UK) and separately propagated in de Man-Rogosa-Sharpe (MRS) broth (CM 0359, Oxoid Ltd, Hampshire, UK) at 30 ± 3 °C for 48 h before their use for feed fermentation.

### 2.2. Fermentation of the Local Feed Stock

**Figure 1:**
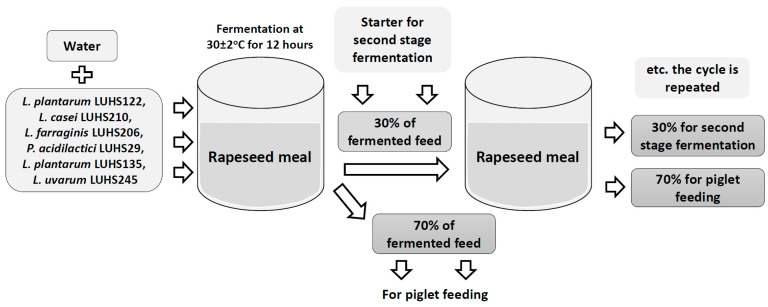
Scheme of fermented feed preparation.

The rapeseed meal (composition: crude protein—19.00%, crude fiber—3.15%, crude oil and fats—6.51%, lysine—1.45%, methionine—0.55%, tryptophan—0.26%, threonine—0.93%, Ca—0.90%, total P—0.59%, and Na—0.20%), water, and LAB strains (equal parts of each strain by volume) suspension (3% from dry matter of feed mass, v/m), containing 8.9 log10 colon-forming units (CFU) mL-1, was fermented at 30 ± 3 °C for 12 h. The final moisture content of the feed was 60 g/100 g. The moisture content was determined by drying the samples at 103 ± 2°C to a constant weight [24]. The whole fermented feed mass (100%) was divided in two parts (30% and 70%, by mass): 70% of the fermented feed was used for piglet feeding, while 30% of fermented feed was used as a starter for additional feed fermentation cycles (Figure 1). Non-fermented rapeseed meal samples were analyzed as the control. Fermented samples were analyzed every week (six weeks) to compare pH and microbiological parameters. 

### 2.3. Evaluation of Fermented Feed pH and Microbiological Parameters

The pH of rapeseed samples was measured using a pH electrode (PP-15; Sartorius, Goettingen, Germany). The microbiological parameters were evaluated according to methods described by Bartkiene et al. [26]. De Man, Rogosa and Sharpe (MRS) agar was used to analyze the LAB count; Violet Red Bile Glucose (VRBG) agar (Oxoid Ltd., Basingstoke, UK) was used to analyze the total enterobacteria count (TEC); Plate Count Agar (Biolife Italiana Srl, Milan, Italy) was used to determine the total count of aerobic and facultative anaerobic bacteria (TBC); and Dichloran Rose Bengal Chloramphenicol (DRBC) agar (Liofilchem, Milan, Italy) was used to analyze yeast/mold (Y/M) count in rapeseed meal samples. The number of microorganisms was counted and expressed as log10 of colony-forming units per gram (CFU/g). All results are expressed as the mean of three determinations.

### 2.4. Animals and Housing 

All animal procedures were conducted according to the EU Directive of the European Parliament and of Council from 22 September 2010 [27] on the protection of animals used for scientific purposes and Requirements for the Keeping, Maintenance, and Use of Animals Intended for Science and Education Purposes, approved by the order of the Lithuanian Director of the State Food and Veterinary Service [28]. The study was conducted at a pig farm in the Klaipeda district (Kontvainiai, Lithuania) and at the Institute of Animal Rearing Technologies, Lithuanian University of Health Sciences (Kaunas, Lithuania). A 36-day experiment was conducted using 25-day-old 200 Large White / Norwegian Landrace (LW/NL) piglets (100 piglets in each group). The trial started with piglets at initial body weight of 6.9 kg–7.0 kg in both (control and treated) groups. The diet of piglets before trial was composed of crude protein—19.09%, crude fiber—3.01%, crude fats—5.98%, av. lysine—1.55%, av. methionine—0.67%, av. tryptophan—0.25%, av. threonine—0.98%, Ca—0.86% and total P—0.62%. The weaner piglets were kept in a section with two climate zones. The first had a heated concrete floor (36 °C) with a roof over it, while the second had plastic piglet floors and optimally ventilated air and temperature for the active period. Drinking water and compound liquid feed were available ad libitum throughout the trial. Antibiotic treatment was not applied.

### 2.5. Experimental Design and Diets 

The piglets were distributed into two groups. Two dietary treatments were compared: (i) a non-fermented basal diet with extruded soybeans and (ii) a fermented basal diet with rapeseed meal. Fermented feed comprised 500 g/kg of the total feed (corresponding to 25% rapeseed meal in treated group diet) it was included in the diet of the treated group beginning at day 25 of life until day 61. Both animal groups were fed with wet feed (water and feed ratio 3/1), equipment used for feeding was WEDA (Dammann & Westerkamp GmbH, Germany).

The piglets’ growth performance was evaluated by testing all 100 piglets from each group; other piglet parameters were evaluated by testing 10 piglets from each group. The basal feed was formulated according to the nutritional requirements prescribed in the Nutrient Requirements of Swine [29]. The feed composition and nutritional value are shown in Table 1. Dietary contents were analyzed according to the AOAC recommendations [30]. 

### 2.6. Metagenomics and Microbial Profiling Analysis

Before the experiment, feces from 10 piglets from the 25-day-old control and treated groups were collected. The DNA from each sample was kept in DNA/RNA Shield (1:10 dilution; R1100-250, Zymo Research, USA) at −70 °C before DNA extraction. DNA was extracted with a fecal DNA MiniPrep kit (D6010, Zymo Research, Irvine, CA, USA). Library preparation, metagenomic sequencing, and taxonomic characterization of reads were performed as described previously [31]. ZymoBIOMICS Microbial Community Standard (D6300, Zymo Research, Murphy Ave, Irvine, CA, USA) was used as a microbiome profiling quality control. The results of taxonomic classification were visualized using the interactive online platform https://genome-explorer.com.

### 2.7. Microbiological Analysis of Fecal Samples

The piglets’ fecal samples were collected before and after the experiment, stored in vials (+4 °C) with a transport medium (Faecal Enteric Plus, Oxoid, Basingstoke, UK), and analyzed on the same day. Evaluation of the microbiological parameters (LAB, TBC, TEC, and Y/M counts) was performed according to methods described by Zavistanaviciute et al. [32]. 

### 2.8. Blood Analysis

Piglets were bled from the jugular vein into vacuum blood tubes (BD Vacutainer, United Kingdom) before the morning feeding. Tubes with clot activator were used for biochemical examination. Blood biochemical variables were evaluated before and after the experiment (on days 25 and 61 of the piglets’ life). The parameters included aspartate aminotransferase (AST), alanine aminotransferase (ALT), cholesterol (mmol), high-density lipoprotein cholesterol (HDL-C), low-density lipoprotein (LDL) cholesterol, triglycerides (TG), total protein (TP), albumin (ALB), phosphorus (IP), magnesium (Mg), potassium (K), sodium (Na), triiodothyronine (T3), thyroxine (T4), immunoglobulin IgG, vitamin B12, albumin (ALB), total protein (TP), iron (Fe), glucose (GLU), calcium (Ca), creatinine analyzed by the Jaffe method (CREA), alkaline phosphatase (AP), thyroid-stimulating hormone (TSH), total bilirubin, and urea. Blood parameters were analyzed with an automatic biochemistry analyzer in the accredited laboratory “Anteja” (Klaipeda, Lithuania). 

### 2.9. Evaluation of the Piglets’ Growth Performance 

Group body weight (BW) gain was recorded on days 32, 39, 46, 53, and 61 of age using an electronic weighing system (model type: IT1000, SysTec GmbH, Bergheim, Germany). The feed conversion ratio (FCR) was calculated from feed intake (87% of dry matter) and BW gain, which was recorded on the same days as BW gain using a WEDA (Dammann & Westerkamp GmbH, Germany) automated feeding system that has an electronic flowmeter and weighing system.

### 2.10. Analysis of Ammonia Emission 

Analysis of ammonia emission was conducted according to the method outlined in the Environmental Protection Document LAND 88-2009, approved by the Nr. D1-862 order (31 12 2009) of the Lithuanian Minister of Environment [33]. Ammonia concentration in the air was analyzed by the accredited laboratory “Labtesta” (Kretinga, Lithuania). Air samples were taken on the first and last days of experiment, in (i) the piglets fed with soya meal sector and (ii) the piglets fed with fermented rapeseed meal sector.

### 2.11. Statistical Analysis

In order to evaluate the influence of fermentation on feed characteristics, data were subjected to analysis of variance (ANOVA) and paired t-test column statistics. All feed sample analytical experiments were performed in triplicate (*n* = 3). ANOVA was also performed to assess the effects of treatment with fermented feed on piglet parameters. Data were subjected to two-way ANOVA using statistical package SPSS for Windows (Ver.15.0, SPSS, Chicago, IL, USA). Baseline measurements were used as covariates to take the experimental conditions into account. The mean values were compared using Duncan’s multiple range test with significance level defined at *p* ≤ 0.05. In the tables, the results are presented as mean values with pooled standard errors (*n* = 10). Differences in bacterial genera between the groups at the end of experiment were assessed using the Z-test calculator for two population proportions (Social Science Statistics, socscistatistics.com, 2019). Statistical comparisons were considered significant when *p* ≤ 0.05.

## 3. Results and Discussion

### 3.1. Fermented Feed Characteristics 

**Figure 2:**
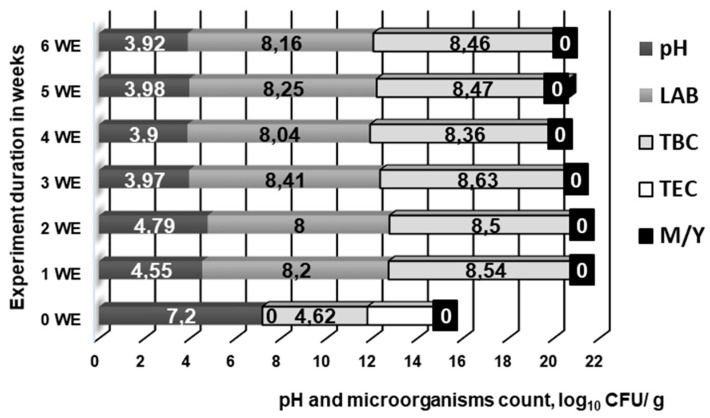
Microbial and pH changes during the feed fermentation process. The data are expressed as the mean ± standard deviation (*n* = 3). The data were statistically compared with a paired t-test and column statistics; *p* ≤ 0.05 was considered significant. Abbreviation: CFU—colony-forming unit.

Microbiological parameters and changes in feed pH during the six weeks of fermentation are shown in Figure 2. In non-fermented feed, Y/M counts were not established; however, LAB, TBC, and TEC counts were 2.3, 4.6, and 3.4 log10 CFU/g, respectively (Figure 2). In fermented feed during the six-week period, the TEC was not established; in addition, the average LAB count was 8.2 ± 0.2 log10 CFU/g, and the average TBC count was 8.5 ± 0.1 log10 CFU/g. The fermented feed pH during the two first weeks was, on average, 4.67 ± 0.17 (Figure 2). However, after three weeks and until the end of the experiment, the pH was reduced; the average was 3.94 ± 0.04. Feed fermentation is associated with improved nutritional value, a high number of viable LAB, and lower pH, as well as a high concentration of organic acids [15,34]. Fermentation protects feed from spoilage and non-desirable (pathogenic and opportunistic) microorganism contamination, factors that ensure biosafety of the biomodified stock [35]. Feed fermentation is a common practice in pig farms [15]. Many factors influence the fermentation process (microorganisms, substrates, moisture content, environmental conditions, etc.). However, complex compounds are metabolized into simpler forms [23]. The types of microorganisms and their characteristics, as well as the fermentation conditions, will result in the formation of different final metabolites, such as lactic acid, bacteriocins, ethanol, etc., because different microorganisms may react distinctly to specific substrates and conditions [35]. In a previous study, the pH of fermented maize kernels decreased from 5.5 to 4.2, and coliform bacteria, TEC, and Y/M decreased from, on average, 6.0 to 3.0 log10 CFU/g, and LAB counts increased to 8.2 log10 CFU/g [36]. Higher LAB counts, lower pH, and a reduced non-desirable microorganism count render fermented feeds beneficial for healthy gut functions [37]. In addition to the above-mentioned fermented feed characteristics, fermentation can decrease mycotoxin content in feedstuffs [38]. In this study, during the six-week feed fermentation using the *L. plantarum* LUHS122, *L. casei* LUHS210, *L. farraginis* LUHS206, *P. acidilactici* LUHS29, *L. plantarum* LUHS135, and *L. uvarum* LUHS245 strain combination, the TEC was almost zero and the LAB count was greater than 8.0 log10 CFU/g. In addition, the pH was, on average, 4.0. In order to avoid losses of essential nutrients in fermented feeds, one study suggested fermenting just the grain fraction (before incorporation essential nutrients) instead of the whole diet [39]. In our study, the rapeseed meal fraction was fermented, and additional essential nutrients were not lost. Finally, fermentation with the above-mentioned LAB combination is suitable for rapeseed meal biomodification.

### 3.2. Microbial Profiles of Pig Feces 

**Figure 3:**
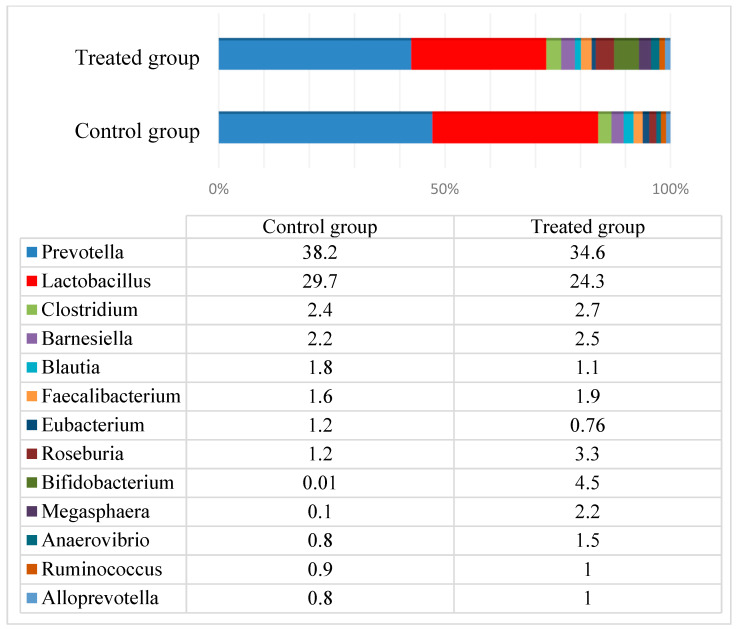
The total microbiota composition in pigs’ feces before the experiment.

The total microbiota composition in pig feces before the experiment is presented in Figure 3. The microbial profiles in both piglet groups before the experiment were very similar: *Prevotella* (34.6–38.2%) and *Lactobacillus* (24.3–29.7%) were most prevalent and represented approximately 60% of the total microbiota. The third most prevalent genus in both groups was *Clostridium*, with a prevalence of only 2.4–2.7%. The other genera included *Barnesiella* (2.2–2.5%), *Faecalibacterium* (1.6–1.9%), *Blautia* (1.1–1.8%), and some other ordinary bacterial genera that comprise the normal microbiome in pigs. The total number of reads was 37,151 and 36,543 in the control and treated groups, respectively. At the end of the experiment, the total number of reads was 34,833 and 37,928 in the control and treated groups, respectively.

**Figure 4:**
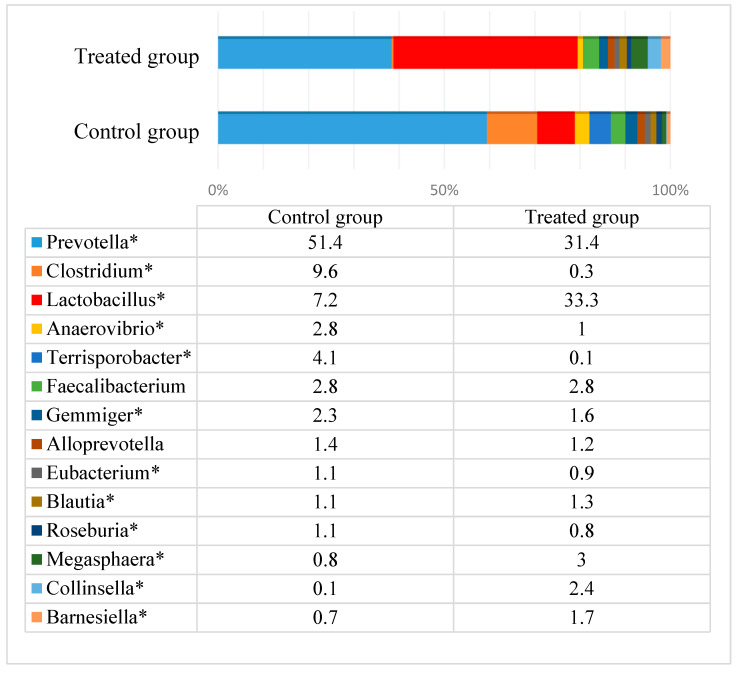
The most prevalent bacterial species (a prevalence of at least 1% from all bacteria in the control or treated group) in the pigs’ feces after the experiment (day 61). * Significant differences in the specific genera between the groups (*p* ≤ 0.05).

After the experiment, the microbial composition significantly changed between the groups (Figure 4). The most obvious change was associated with the decreased number of *Lactobacillus* in the control group (7.2%), whereas in the treated group, it remained high and reached one third of the total amount of bacteria. In other words, *Lactobacillus* was more than four times higher in the treated compared to the control group. There were also changes with some other genera, including *Collinsella* and *Megaspahera*, which had significantly higher prevalence in the treated group, as well as *Terrisporobacter* and *Anaerovibrio*, of which the prevalence was higher in the control group. In pigs, the abundance of *Collinsella* positively corelates with apparent neutral detergent fiber (NDF) and acid detergent fiber (ADF) digestibility [40]. *Megasphaera elsdenii* is known to be the cause of the inhibition of the pathogenic bacteria *Brachyspyra hyodysenteriae* in the colon of pigs when they are fed fructan-rich diets [41]. *M. elsdenii* was the third most prevalent species in the gut of the treated group. The other most prevalent species in this group were *Lactobacillus amylovorus* and *Prevotella copri*. More differences at the species level are presented in Supplementary Files 1 and 2. According to the obtained data, the diet with treated feed had a positive impact on fecal microbiota in pigs, particularly due to its ability to maintain a high number of lactobacilli. *Lactobacillus* bacteria are probiotics: They help reduce the amount of pathogenic bacteria in the gut and provide a potential alternative to antibiotic strategies [42]. This factor is crucial in the context of fighting antimicrobial resistant bacteria. 

### 3.3. Influence of Fermented Feed on LAB, TEC, and Y/M Count in Piglets’ Feces 

Microbiological parameters of the piglet feces (LAB, TBC, TEC, and Y/M counts) are presented in Table 2. There were no significant differences between the LAB count in the control and treated group feces. When comparing the control and treated group feces of the 25-day-old piglets, the TBC was significantly different (*p* = 0.002); specifically, there was a 1.3 log10 CFU/g higher TBC in the treated compared to the control group feces. When comparing the TEC in the treated group feces of the 25- and 61-day-old piglets, it was significantly lower at the end of experiment (by 0.5 log10 CFU/g lower, *p* = 0.013). However, when comparing the control and treated group samples at the end of experiment, TEC was significantly lower in the control group (by 0.5 log10 CFU/g, *p* = 0.013). Further, when comparing the beginning and the end of experiment, the Y/M count in the control and treated group feces at day 61 were significantly lower (*p* = 0.002 and *p* = 0.013, respectively). However, when comparing the control and treated group samples from the 61-day-old piglets, the Y/M count in treated group samples was significantly lower compared to the control group (by 0.71 log10 CFU/g, *p* = 0.002). Promoting intestinal maturation during the early-life period has great potential to improve the growth, development, and disease resistance of neonatal mammals [43]. An early intervention from the gut microbiota may be promising to improve intestinal microbial ecology [44,45,46,47]. LAB also play a key role in disease prevention [48]. *Lactobacillus* are used as probiotics after weaning because they are commensal bacteria that regulate gut immune function, maintain the balance of gut microbiota, and reduce inflammatory responses [49,50,51,52]. In the present study, there were no significant differences between the LAB count in control and treated group feces; however, a previous study indicated that nonviable LAB can also positively influence health parameters [53]. Further, in the current study, TEC was higher in the treated piglets’ feces at the end of experiment. *Enterobacteriaceae* are natural microflora of human and animal guts. They can cause enteric diseases or remain as commensal organisms, and only a small group of species are considered to be strict pathogens [54]. In this study, fermented feed reduced the Y/M count in the treated piglets’ feces. Our previous studies showed that the same LAB combination inhibits mold growth *in vitro* [26,38]. Finally, more parameters should be considered to evaluate the influence of fermented feed on piglets’ health and growth performance. Additionally, according to metagenomics analysis of the piglets’ feces, the diet using fermented rapeseed meal positively impacted fecal microbiota in pigs, particularly due to its ability to maintain a high number of lactobacilli. However, there were no significant differences when comparing viable LAB cells in feces.

### 3.4. Piglet Blood Parameters 

Table 3 presents the piglet blood parameters. There was a significantly lower AST concentration in 61-day-old treated group piglets (*p* = 0.039) compared to 25-day-old treated pigs (beginning of the experiment). However, when comparing the control and treated group samples at the end of experiment, AST was significantly lower in the control group (*p* = 0.004). ALT was significantly higher in treated group blood samples at the beginning (*p* = 0.01) and the end (*p* = 0.02) of the experiment when compared to the control group. Further, ALT was significantly higher at the end compared to the beginning of the experiment (*p* = 0.0001). 

At the beginning of the experiment, cholesterol (Chol) was significantly higher in the control compared to the treated group (*p* = 0.0001); however, at the end of experiment, there was no significant difference between the groups. HDL-C in all the cases was higher in the treated group, but LDL-C concentrations were not different between the groups at the end of the experiment. Notably, at the beginning of experiment, LDL-C was significantly higher in the control group (*p* = 0.009). At the end of experiment, TG was significantly higher in the treated compared to the control group (*p* = 0.005). When comparing samples at the beginning and the end of experiment, TG was significantly higher in the control group (*p* = 0.012). 

There were no differences between the groups at the end of the experiment in TP, ALB, and IgG, but when comparing both groups at the beginning and at the end of experiment, IgG was significantly higher at the end of experiment in the control (*p* = 0.037) and treated (*p* = 0.031) groups. T3 and T4 concentrations at the end of experiment were significantly lower in the treated compared to the control group samples (*p* = 0.0001). There were no differences in the GLU concentration between the groups. 

With regard to the tested micro- and macroelements in piglets’ blood samples, in all the cases, there was a significantly higher IP concentration in the control group (at the beginning and at the end of experiment). There were the same tendencies with Mg and Ca in the control group; both were significantly higher at the end of experiment. There were no significant differences in K, Na, and Fe at the end of experiment between the control and treated group blood samples. The vitamin B12 concentration was significantly higher in the control group; however, for treated group blood samples, at the end of experiment, vitamin B12 was significantly increased compared to the beginning of the experiment (*p* = 0.021). There were no significant differences at the end of experiment between the control and treated groups for CREA, AP, urea, TSH, or total bilirubin. 

A previous study indicated that the addition of fermented rapeseed meal in the piglet diet significantly influences ALT, AST, AP, and HDL-C levels [55]. According to other authors, fermented feed has a significant influence on the higher proportion of the HDL-C fraction because fermented feed is linked to normal thyroid function, which stimulates lipid metabolism [55,56]. Also, the above-mentioned response is associated with the high intake of viable LAB, which are the main microorganisms in fermented feed and can mediate the changes in the lipid profile [56]. Fermented rapeseed meal in the piglet diet can also lead to better mineral availability, increase production efficiency, and improve the hematological profile [57]. Finally, by changing extruded soya to cheaper rapeseed meal and applying the fermentation model, it is possible to feed piglets without any undesirable changes in the blood profile.

### 3.5. Piglets’ Growth Performance 

**Figure 5:**
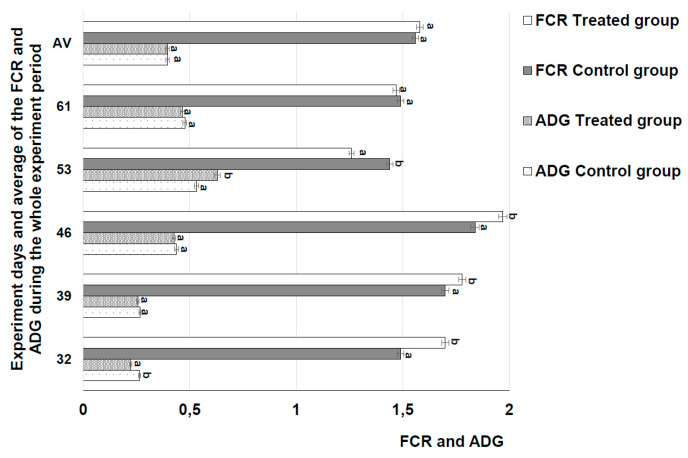
Average daily gain (ADG) and feed conversion ratio (FCR) of the piglets. The data are expressed as the mean ± standard deviation (*n* = 100). The data were statistically compared with a paired t-test and column statistics. Means followed by different letters (a,b) are significantly different (*p* ≤ 0.05).

The piglets’ average daily gain (ADG) and FCR are shown in Figure 5. When comparing ADG and FCR of the control and treated groups from day 32 to 61 of the experiment, there were no significant differences. Until six weeks of age, piglets have a limited ability to digest food, higher immune system sensitivity [58], and diarrhea caused by stress [59]. Fermented feed reportedly reduces diarrhea and improves the piglets’ health parameters [60]. The uses of fermented feed can be a strategy to reduce antimicrobial growth promoters in farms, positively affect gut health, improve productivity, and reduce feed price [61,62]. In addition, fermented feed provides desirable functional microbes [63]. Fermentation also reduces antinutritional factors in feed [64]. Koo et al. [65] reported that the addition of fermented barley enhances weaner piglets’ gut health and increases immune responses and gut bacteria composition. According to Cheng et al. [66] and Xu et al. [67], fermented soybean meal has a positive influence on the growth performance of weaned piglets. Furthermore, new compounds formed in feed during fermentation can increase the growth performance of weaned piglets and growing pigs [67]. In our study, there were no significant differences in ADG and FCR between the control and treated groups. However, it should be mentioned that fermentation allowed the switch from expensive extruded soya to more cost effective rapseed meal while attaining the same ADG and FCR. Finally, fermentation with the selected LAB combination led to more economical feeding: A local feed stock was used rather than imported soya products.

### 3.6. Influence of Fermented Feed on Ammonia Emission 

**Figure 6:**
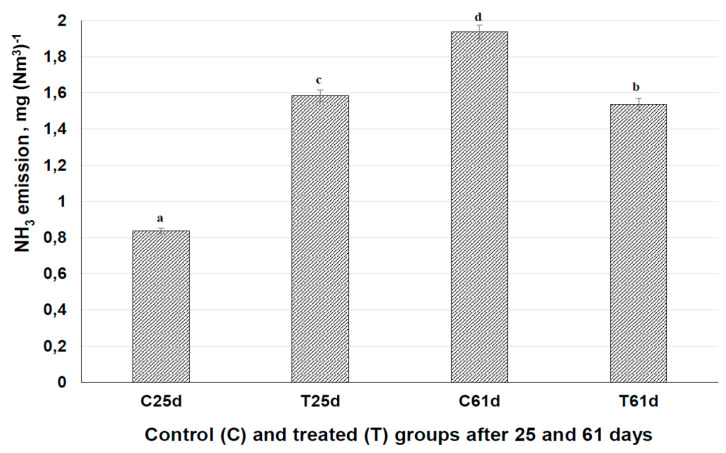
Ammonia (NH3) emission in farm sections with piglets fed non-fermented and fermented feeds. The data are expressed as the mean ± standard deviation (*n* = 3). The data were statistically compared with a paired t-test and column statistics. Means followed by different letters (a,b,c,d) are significantly different (*p* ≤ 0.05).

Ammonia emission at the different sections where the control and treated piglets were kept is shown in Figure 6. There was significantly higher ammonia emission at the beginning of experiment in the treated group section (1.9 times higher compared with the control group section). However, at the end of experiment, in the treated group section, ammonia emission was significantly lower (by 20.6%). Ammonia is not just an ecological problem; it also has a negative influence on animal health and can cause infectious diseases [68]. It is very important to reduce ammonia emissions in pig farms. This endeavor can promote animal growth, improve the economic performance of farms, and reduce environmental pollution. The main factors associated with ammonia emissions are the fecal sewage treatment system [69], feeding technology, and the housing system [70]. The dietary composition can contribute as one of the main factors [71]. In our study, changing from extruded soya to fermented rapeseed meal significantly reduced ammonia emission in the tested piglets section. This fermentation strategy leads to effective use of local stocks and reduced dependence on imported soya, as well as higher economical effectiveness. The reduction of ammonia by modifying dietary composition is considered economical and feasible [66]. The significant ammonia emission reduction noted with the biomodified rapeseed compared with extruded soya meal underscores the lower dependence on imported soya products. 

## 4. Conclusions

Our data recommend the use of a combination of newly isolated antimicrobial-characteristic-possessing LAB starters (L. plantarum LUHS122, L. casei LUHS210, L. farraginis LUHS206, P. acidilactici LUHS29, L. plantarum LUHS135, and L. uvarum LUHS245) for rapeseed meal fermentation (viable LAB count in fermented feed 8.5 ± 0.1 log10 CFU/g, pH 3.94 ± 0.04). In addition, changing from an extruded soya to fermented rapeseed meal in a piglet feed recipe led to desirable changes in piglets’ fecal microbiota (more than four times higher Lactobacillus count than in a control group) and significantly lower ammonia emission (20.6% reduction). Finally, by changing extruded soya to less expensive rapeseed meal and applying the fermentation model described in this study, it is possible to feed piglets without any undesirable changes in health and growth performance. However, it should be mentioned that a broader spectrum of the gas emissions should be tested to indicate the real impact of the tested diet on the environment.

## Figures and Tables

**Table 1:** Diet composition.

Ingredients (%)	Control Group	Treated Group
Barley	38.40	33.25
Rapeseed meal	-	25.00
Wheat	32.12	25.02
Soya beans (extruded)	9.30	-
Potato protein	5.00	2.00
Soybean protein concentrate	2.00	-
Whey powder	5.80	5.80
Sunflower oil	2.72	4.51
Limestone	1.48	1.1
NaCl	0.38	0.35
Monocalcium phosphate	0.33	0.41
L-Lysine sulfate	0.87	1.1
DL-Methionine	0.25	0.16
Acidal NC (formic and acetic acids)	0.30	0.30
^1^Vitamins and trace elements (premix)	1.00	1.00
Bredol 683	0.05	0.00
Nutritional value		
ME swine (MJ/kg)	13.86	13.95
Crude protein (%)	19.00	19.00
Crude fat (%)	6.51	6.51
Crude fiber (%)	3.15	5.14
Lysine (%)	1.45	1.45
Methionine (%)	0.55	0.55
Threonine (%)	0.93	0.94
Tryptophan (%)	0.26	0.25
Methionine + Cystine (%)	0.87	0.88
Ca (%)	0.90	0.90
Total P (%)	0.59	0.62
Available P (%)	0.37	0.38
Na (%)	0.20	0.21

ME—metabolizable energy ^1^Composition of premix per 1 kg of feed: Vitamin A—18,180 IU; vitamin D3—2040 IU; vitamin E—161 mg/kg; vitamin K3—5.03 mg; thiamine—3.64 mg; riboflavin—9.16 mg; choline chloride—404 mg; pyridoxine—4.60 mg; vitamin B12—0.05 mg; niacin—41 mg; pantothenic acid—22.85 mg; folic acid—1.85 mg; biotin—0.21 mg; Fe—152 mg; Cu—101 mg; Zn—91 mg; Mn—80 mg; I—0.81 mg; Co—0.53 mg; Se—0.30 mg.

**Table 2:** Microbiological parameters in feces from 25- and 61-day-old pigs.

Microbiological Parameters (log_10_ CFU/g)	Day	Treatments		*p*
		C	T	Day × Treatment Interaction
LAB	Baseline	7.8 ± 0.3 ^Aa^	8.3 ± 0.1^Ab^	0.0001
	61	6.2 ± 0.1 ^Bb^	5.2 ± 0.1 ^Ba^	
TBC	Baseline	7.1 ± 0.2 ^Aa^	8.4 ± 0.1 ^Ab^	0.0001
	61	6.4 ± 0.1 ^Ba^	6.4 ± 0.1 ^Ba^	
TEC	Baseline	7.2 ± 0.1 ^Ba^	7.4 ± 0.1 ^Ba^	0.081
	61	6.4 ± 0.2 ^Aa^	6.9 ± 0.1 ^Ab^	
Y/F	Baseline	6.7 ± 0.1 ^Bb^	6.2 ± 0.1 ^Ba^	0.122
	61	6.4 ± 0.1 ^Ab^	5.7 ± 0.1 ^Aa^	

LAB—lactic acid bacteria; TBC— total bacteria count; TEC—total enterobacteria count; Y/M—yeast/mold count; CFU—colony-forming units; C—control group, fed with the basal diet; T—treated group, fed with the fermented feed; 25d—25-day-old piglets; 61d—61-day-old piglets. ^A,B^ different capitals indicate significant time-related differences (*p* < 0.05); ^a,b^ different letters indicate differences among treatments (*p* < 0.05) Data are presented as mean ± SE (*n* = 10/group).Baseline measurements were done on d 25, before the start of the feeding experiment.

**Table 3:** Blood parameters of the piglets.

Blood Parameters	Day	Treatments		*p*
		C	T	Day × Treatment Interaction
Aspartate aminotransferase (AST), U/L	Baseline	29.4 ± 3.4 ^Aa^	51.4 ± 11.2 ^Ab^	0.204
	61	34.0 ± 6.1 ^Aa^	44.0 ± 7.2 ^Aa^	
Alanine aminotransferase (ALT), U/L	Baseline	48.4 ± 6.8 ^Aa^	53.2 ± 11.6 ^Aa^	0.647
	61	76.2 ± 11.8 ^Ba^	87.0 ± 12.5 ^Ba^	
Cholesterol (Chol), mmol/L	Baseline	1.63 ± 0.21 ^Aa^	1.88 ± 0.54 ^Aa^	0.943
	61	2.06 ± 0.21 ^Ba^	2.34 ± 0.35 ^Aa^	
High-density lipoprotein cholesterol (HDL-C), mmol/L	Baseline	0.714 ± 0.134 ^Aa^	0.898 ± 0.201 ^Aa^	0.976
	61	0.840 ± 0.134 ^Aa^	1.03 ± 0.18 ^Aa^	
Low-density lipoprotein cholesterol (LDL-C), mmol/L	Baseline	0.758 ± 0.086^Aa^	0.814 ± 0.329 ^Aa^	0.987
	61	0.980 ± 0.123 ^Ba^	1.032 ± 0.173 ^Aa^	
Triglycerides (TG), mmol/L	Baseline	0.360 ± 0.130 ^Aa^	0.366 ± 0.063 ^Aa^	0.245
	61	0.466 ± 0.092 ^Aa^	0.620 ± 0.111 ^Ba^	
Total protein (TP), g/L	Baseline	46.2 ± 2.3 ^Aa^	44.2 ± 2.1 ^Aa^	0.391
	61	51.8 ± 2.8 ^Ba^	52.8 ± 3.9 ^Ba^	
Albumin (ALB), g/L	Baseline	30.0 ± 2.1 ^Aa^	32.6 ± 3.1 ^Aa^	0.558
	61	35.8 ± 3.9 ^Aa^	36.2 ± 3.1 ^Aa^	
Immunoglobulin IgG, g/L	Baseline	2.64 ± 0.797^Aa^	2.35 ± 0.705 ^Aa^	0.684
	61	3.73 ± 1.10 ^Aa^	3.05 ± 0.467 ^Aa^	
Triiodothyronine (T3), nmol/L	Baseline	1.21 ± 0.297 ^Aa^	1.30 ± 0.315 ^Aa^	0.046
	61	2.14 ± 0.128 ^Bb^	1.59 ± 0.143 ^Aa^	
Thyroxine (T4), µ d/L	Baseline	4.50 ± 0.424 ^Ab^	3.50 ± 0.346 ^Aa^	0.047
	61	4.80 ± 0.230 ^Ab^	2.92 ± 0.268 ^Ab^	
Glucose (GLU), nmol/L	Baseline	5.84 ± 0.737 ^Aa^	6.12 ± 0.259 ^Aa^	0.971
	61	5.74 ± 0.503 ^Aa^	6.08 ± 0.286 ^Aa^	
Phosphorus (IP), mmol/L	Baseline	2.94 ± 0.327 ^Aa^	2.61 ± 0.371 ^Aa^	0.737
	61	3.50 ± 0.144 ^Ba^	3.28 ± 0.183 ^Ba^	
Magnesium (Mg), mmol/L	Baseline	1.02 ± 0.117 ^Aa^	0.996 ± 0.106 ^Aa^	0.429
	61	1.07 ± 0.054 ^Aa^	0.960 ± 0.0590 ^Aa^	
Potassium (K)	Baseline	4.96 ± 0.427 ^Aa^	4.65 ± 0.298 ^Aa^	0.368
	61	5.81 ± 0.35 ^Ba^	4.96 ± 0.747 ^Aa^	
Sodium (Na)	Baseline	143.4 ± 3.05 ^Aa^	144.0 ± 1.0 ^Aa^	0.591
	61	147.2 ± 0.837 ^Aa^	146.6 ± 1.67 ^Aa^	
Iron (Fe), µmol/L	Baseline	23.6 ± 5.9 ^Aa^	31.5 ± 3.9 ^Aa^	0.195
	61	28.1 ± 2.2 ^Aa^	47.1 ± 11.4 ^Bb^	
Calcium (Ca), nmol/L	Baseline	2.60 ± 0.217 ^Aa^	2.71 ± 0.035 ^Aa^	0.261
	61	2.87 ± 0.129 ^Aa^	2.79 ± 0.096 ^Aa^	
Vitamin B12, pmol/L	Baseline	142.2 ± 32.32^Ab^	78.2 ± 19.1 ^Aa^	0.270
	61	214.6 ± 64.8 ^Ab^	94.2 ± 34.4 ^Aa^	
Creatinine (CREA), µmol/L	Baseline	64.2 ± 11.7 ^Aa^	78.8 ± 17.5 ^Ba^	0.120
	61	57.4 ± 3.7 ^Aa^	48.2 ± 10.2 ^Aa^	
Alkaline phosphatase (AP), U/L	Baseline	336.2 ± 132.9 ^Aa^	408.6 ± 165.5 ^Aa^	0.502
	61	263.6 ± 83.8 ^Aa^	242.6 ± 29.9 ^Aa^	
Urea, mmol/L	Baseline	2.36 ± 0.49 ^Aa^	2.64 ± 0.624 ^Aa^	0.207
	61	2.02 ± 0.14 ^Aa^	3.19 ± 0.778 ^Ab^	
Thyroid-stimulating hormone (TSH)	Baseline	0.02 ± 0.005 ^Aa^	0.021 ± 0.002 ^Aa^	0.666
	61	0.021 ± 0.01 ^Aa^	0.023 ± 0.012 ^Aa^	
Total bilirubin (pmol/L)	Baseline	˂2	˂2	-
	61	˂2	˂2	

C—control group, fed with the basal diet; T—treated group, fed with the fermented feed; 25d—25-day-old piglets; 61d—61-day-old piglets. ^A,B^ different capitals indicate significant time-related differences (*p* < 0.05); ^a,b^ different letters indicate differences among treatments (*p* < 0.05). Data are presented as mean ± SE (*n* = 10/group) Baseline measurements were done on d 25, before the start of the feeding experiment.

## Supplementary Materials

The following are available online at https://www.mdpi.com/2076-2615/10/5/783/s1, Table S1: Control group species; Table S2: Experimental group species after experiment.
