# Supplementary material for: Pigs’ Feed Fermentation Model with Antimicrobial Lactic Acid Bacteria Strains Combination by Changing Extruded Soya to Biomodified Local Feed Stock
Source: Animals (Basel). 2020 Apr 30;10(5):783. doi: 10.3390/ani10050783 (PMC7277722; doi:10.3390/ani10050783)
Supplement: Supplementary file 1 [file animals-10-00783-s001.pdf]

**Table S1 Control group species**

| Control group after experiment, species | Number of reads | Relative abundance |
|---|---|---|
| Prevotella copri | 14483 | 41.58% |
| Clostridium cellulovorans | 2471 | 7.09% |
| Lactobacillus amylovorus | 1741 | 5% |
| Terrisporobacter glycolicus | 1403 | 4.03% |
| Prevotella stercorea | 1126 | 3.23% |
| Faecalibacterium prausnitzii | 972 | 2.79% |
| Anaerovibrio lipolyticus | 970 | 2.78% |
| Gemmiger formicilis | 787 | 2.26% |
| Unclassified | 748 | 2.15% |
| Prevotella oralis | 637 | 1.83% |
| Prevotella oris | 586 | 1.68% |
| Alloprevotella rava | 470 | 1.35% |
| Clostridium celatum | 435 | 1.25% |
| Prevotella brevis | 368 | 1.06% |
| Eubacterium rectale | 308 | 0.88% |
| Roseburia faecis | 303 | 0.87% |
| Megasphaera elsdenii | 272 | 0.78% |
| Butyricicoccus pullicaecorum | 231 | 0.66% |
| Prevotella histicola | 225 | 0.65% |
| Barnesiella intestinihominis | 215 | 0.62% |
| Lactobacillus reuteri | 172 | 0.49% |
| Blautia wexlerae | 155 | 0.44% |
| Phascolarctobacterium succinatutens | 148 | 0.42% |
| Sporobacter termitidis | 135 | 0.39% |
| Intestinibacter bartlettii | 135 | 0.39% |
| Prevotella dentalis | 131 | 0.38% |
| Lactobacillus pontis | 129 | 0.37% |
| Paraprevotella clara | 118 | 0.34% |
| Fusicatenibacter saccharivorans | 111 | 0.32% |
| Romboutsia sedimentorum | 109 | 0.31% |
| Eubacterium ramulus | 104 | 0.3% |
| Eubacterium coprostanoligenes | 101 | 0.29% |
| Clostridium quinii | 92 | 0.26% |
| Lactobacillus crispatus | 86 | 0.25% |
| Lactobacillus panis | 85 | 0.24% |
| Catenibacterium mitsuokai | 84 | 0.24% |
| Oscillospira guilliermondii | 81 | 0.23% |
| Bacteroidales oral | 78 | 0.22% |
| Blautia obeum | 67 | 0.19% |
| Lactobacillus kitasatonis | 67 | 0.19% |
| Eubacterium hallii | 65 | 0.19% |
| Ruminococcus faecis | 63 | 0.18% |
| unclassified Prevotella | 60 | 0.17% |
| cyanobacterium enrichment | 58 | 0.17% |
| Dialister succinatiphilus | 56 | 0.16% |
| Clostridium amylolyticum | 52 | 0.15% |
| Campylobacter lanienae | 48 | 0.14% |
| Ruminococcus bicirculans | 48 | 0.14% |

| | | |
|---|---|---|
| unclassified Bacteroidales | 47 | 0.13% |

| | | |
|---|---|---|
| Fournierella massiliensis | 46 | 0.13% |
| Solobacterium moorei | 46 | 0.13% |
| Lactobacillus johnsonii | 46 | 0.13% |
| Murimonas intestini | 44 | 0.13% |
| Ruminiclostridium thermocellum | 44 | 0.13% |
| Flintibacter butyricus | 43 | 0.12% |
| Prevotella salivae | 43 | 0.12% |
| Roseburia inulinivorans | 43 | 0.12% |
| Lactobacillus jensenii | 42 | 0.12% |
| unclassified Turicibacter | 42 | 0.12% |
| Erysipelothrix inopinata | 41 | 0.12% |
| Butyrivibrio fibrisolvens | 40 | 0.11% |
| Clostridium disporicum | 39 | 0.11% |
| unclassified Clostridium | 39 | 0.11% |
| Prevotella genomosp. | 38 | 0.11% |
| Holdemanella biformis | 38 | 0.11% |
| Oscillibacter ruminantium | 38 | 0.11% |
| Turicibacter sanguinis | 36 | 0.1% |
| Prevotella ruminicola | 36 | 0.1% |
| Asaccharospora irregularis | 35 | 0.1% |
| Ruminococcus flavefaciens | 34 | 0.1% |
| Clostridium sartagoforme | 34 | 0.1% |
| Ruminococcus torques | 34 | 0.1% |
| Coprococcus comes | 32 | 0.09% |
| Eubacterium eligens | 32 | 0.09% |
| Prevotella maculosa | 31 | 0.09% |
| Sutterella stercoricanis | 29 | 0.08% |
| Dorea formicigenerans | 29 | 0.08% |
| Mitsuokella jalaludinii | 29 | 0.08% |
| Lactobacillus delbrueckii | 28 | 0.08% |
| Eubacterium desmolans | 28 | 0.08% |
| unclassified Tannerella | 28 | 0.08% |
| unclassified Barnesiella | 27 | 0.08% |
| Clostridium bovipellis | 27 | 0.08% |
| Eubacterium ruminantium | 27 | 0.08% |
| Blautia faecis | 26 | 0.07% |
| Blautia luti | 26 | 0.07% |
| Dorea longicatena | 25 | 0.07% |
| Collinsella aerofaciens | 25 | 0.07% |
| Clostridium aldenense | 25 | 0.07% |
| Hungatella hathewayi | 25 | 0.07% |
| Lachnospira pectinoschiza | 24 | 0.07% |
| Parabacteroides distasonis | 24 | 0.07% |
| Blautia massiliensis | 23 | 0.07% |
| Anaerotaenia torta | 23 | 0.07% |
| Clostridium leptum | 22 | 0.06% |
| Blautia glucerasea | 22 | 0.06% |
| Clostridium saccharolyticum | 21 | 0.06% |
| Coprococcus catus | 21 | 0.06% |
| Ruminococcus bromii | 21 | 0.06% |

| | | |
|---|---|---|
| Coprococcus eutactus | 21 | 0.06% |
| Clostridium cadaveris | 20 | 0.06% |
| unclassified Lachnospiraceae | 20 | 0.06% |
| Holdemania filiformis | 20 | 0.06% |
| Selenomonas ruminantium | 20 | 0.06% |
| Lactobacillus acidophilus | 20 | 0.06% |
| Acetivibrio ethanolgignens | 19 | 0.05% |
| Blautia stercoris | 19 | 0.05% |
| Candidatus Soleaferrea | 19 | 0.05% |
| Prevotella denticola | 18 | 0.05% |
| Clostridium tertium | 18 | 0.05% |
| Clostridium hveragerdense | 18 | 0.05% |
| Oribacterium sinus | 18 | 0.05% |
| Brassicibacter thermophilus | 18 | 0.05% |
| Lactobacillus helveticus | 17 | 0.05% |
| Clostridium chartatabidum | 17 | 0.05% |
| Anaerostipes hadrus | 17 | 0.05% |
| Anaerobacterium chartisolvens | 17 | 0.05% |
| Falcatimonas natans | 16 | 0.05% |
| Roseburia hominis | 16 | 0.05% |
| Succinivibrio dextrinosolvens | 16 | 0.05% |
| Roseburia intestinalis | 15 | 0.04% |
| Agathobacter ruminis | 15 | 0.04% |
| Clostridium chauvoei | 15 | 0.04% |
| Clostridium polysaccharolyticum | 15 | 0.04% |
| Propionispira arcuata | 15 | 0.04% |
| Intestinimonas butyriciproducens | 15 | 0.04% |
| Denitrobacterium detoxificans | 14 | 0.04% |
| Candidatus Dorea | 14 | 0.04% |
| Eubacterium xylanophilum | 14 | 0.04% |
| Gracilibacter thermotolerans | 14 | 0.04% |
| Ruminococcus callidus | 13 | 0.04% |
| unclassified Erysipelotrichaceae | 13 | 0.04% |
| Peptococcus simiae | 13 | 0.04% |
| unclassified Ruminococcaceae | 13 | 0.04% |
| unclassified Clostridiales | 12 | 0.03% |
| Prevotella loescheii | 12 | 0.03% |
| Bifidobacteriaceae genomosp. | 12 | 0.03% |
| Elbe River | 12 | 0.03% |
| Clostridium populeti | 12 | 0.03% |
| Prevotella conceptionensis | 12 | 0.03% |
| Clostridium aurantibutyricum | 12 | 0.03% |
| unclassified Prevotellaceae | 12 | 0.03% |
| unclassified Anaerovibrio | 12 | 0.03% |
| Bacteroides intestinalis | 11 | 0.03% |
| Anaerovorax odorimutans | 11 | 0.03% |
| Olsenella scatoligenes | 11 | 0.03% |
| Candidatus Treponema | 11 | 0.03% |
| Lactobacillus frumenti | 11 | 0.03% |
| Clostridium xylanolyticum | 11 | 0.03% |

| | | |
|---|---|---|
| Clostridium methylpentosum | 11 | 0.03% |
| Eisenbergiella tayi | 10 | 0.03% |
| Ruthenibacterium lactatiformans | 10 | 0.03% |
| Clostridium cellulolyticum | 10 | 0.03% |
| Marvinbryantia formatexigens | 10 | 0.03% |
| Bifidobacterium pseudolongum | 10 | 0.03% |
| Clostridium lavalense | 10 | 0.03% |
| Prevotella paludivivens | 9 | 0.03% |
| Prevotella buccae | 9 | 0.03% |
| Gorbachella massiliensis | 9 | 0.03% |
| Vallitalea pronyensis | 9 | 0.03% |
| Methylocystis rosea | 9 | 0.03% |
| Acidaminococcus fermentans | 9 | 0.03% |
| Clostridium cellobioparum | 9 | 0.03% |
| Herbinix luporum | 9 | 0.03% |
| Blautia producta | 9 | 0.03% |
| Intestinimonas timonensis | 9 | 0.03% |
| unclassified Bacteroides | 9 | 0.03% |
| Prevotella dentasini | 9 | 0.03% |
| Enorma massiliensis | 9 | 0.03% |
| Anaerobium acetethylicum | 9 | 0.03% |
| Selenomonas bovis | 9 | 0.03% |
| Parabacteroides chinchillae | 8 | 0.02% |
| Natranaerovirga pectinivora | 8 | 0.02% |
| Mogibacterium diversum | 8 | 0.02% |
| Eubacterium oxidoreducens | 8 | 0.02% |
| Prevotella bivia | 8 | 0.02% |
| Mobilitalea sibirica | 8 | 0.02% |
| Eubacterium tenue | 8 | 0.02% |
| unclassified Planctomycetales | 8 | 0.02% |
| Clostridium clostridioforme | 8 | 0.02% |
| Acetivibrio cellulolyticus | 8 | 0.02% |
| Intestinimonas massiliensis | 8 | 0.02% |
| unclassified Mollicutes | 8 | 0.02% |
| Treponema porcinum | 7 | 0.02% |
| Bacteroidales genomosp. | 7 | 0.02% |
| Clostridium fimetarium | 7 | 0.02% |
| Eubacteriaceae oral | 7 | 0.02% |
| Faecalitalea cylindroides | 7 | 0.02% |
| Alloprevotella tannerae | 7 | 0.02% |
| unclassified Bulleidia | 7 | 0.02% |
| Subdoligranulum variabile | 7 | 0.02% |
| Clostridium tarantellae | 7 | 0.02% |
| Eubacterium rangiferina | 7 | 0.02% |
| unclassified Candidatus Glomeribacter | 6 | 0.02% |
| Porphyromonas catoniae | 6 | 0.02% |
| Barnesiella viscericola | 6 | 0.02% |
| Paeniclostridium ghonii | 6 | 0.02% |
| Clostridium sphenoides | 6 | 0.02% |
| Parasporobacterium paucivorans | 6 | 0.02% |

| | | |
|---|---|---|
| Selenomonas sputigena | 6 | 0.02% |
| Hallella seregens | 6 | 0.02% |
| Abyssivirga alkaniphila | 6 | 0.02% |
| Clostridium butyricum | 6 | 0.02% |
| Faecalicoccus acidiformans | 6 | 0.02% |
| unclassified Acetivibrio | 6 | 0.02% |
| Clostridium oroticum | 6 | 0.02% |
| Clostridium symbiosum | 6 | 0.02% |
| Terrisporobacter mayombei | 6 | 0.02% |
| Clostridium phoceensis | 6 | 0.02% |
| Lactobacillus fermentum | 6 | 0.02% |
| Clostridium papyrosolvens | 6 | 0.02% |
| Lactobacillus hamsteri | 6 | 0.02% |
| Caloramator quimbayensis | 6 | 0.02% |
| Olsenella profusa | 5 | 0.01% |
| Lutispora thermophila | 5 | 0.01% |
| Propionispira paucivorans | 5 | 0.01% |
| Olsenella umbonata | 5 | 0.01% |
| unclassified Eubacterium | 5 | 0.01% |
| Bacteroides stercoris | 5 | 0.01% |
| Clostridium hiranonis | 5 | 0.01% |
| Lachnospira multipara | 5 | 0.01% |
| Prevotella baroniae | 5 | 0.01% |
| Bacteroides timonensis | 5 | 0.01% |
| Anaerostipes butyraticus | 4 | 0.01% |
| Clostridium botulinum | 4 | 0.01% |
| Ruminococcus albus | 4 | 0.01% |
| unclassified Clostridia | 4 | 0.01% |
| Eubacterium cellulosolvens | 4 | 0.01% |
| unclassified Bacteroidaceae | 4 | 0.01% |
| Ruminococcus lactaris | 4 | 0.01% |
| Lactobacillus rogosae | 4 | 0.01% |
| Desulfovibrio piger | 4 | 0.01% |
| Enterorhabdus mucosicola | 4 | 0.01% |
| unclassified Alloprevotella | 4 | 0.01% |
| Ruminococcus gnavus | 4 | 0.01% |
| Flavonifractor plautii | 4 | 0.01% |
| Prevotella enoeca | 4 | 0.01% |
| Campylobacter hyointestinalis | 4 | 0.01% |
| Anaerocolumna xylanovorans | 4 | 0.01% |
| Olsenella uli | 4 | 0.01% |
| Bacteroides zoogleoformans | 4 | 0.01% |
| Clostridium intestinale | 4 | 0.01% |
| Bacteroides clarus | 4 | 0.01% |
| Mitsuokella multacida | 4 | 0.01% |
| Clostridium fusiformis | 4 | 0.01% |
| Corynebacterium provencense | 3 | 0.01% |
| Clostridium asparagiforme | 3 | 0.01% |
| Asteroleplasma anaerobium | 3 | 0.01% |
| Clostridium celerecrescens | 3 | 0.01% |

| | | |
|---|---|---|
| Lactobacillus secaliphilus | 3 | 0.01% |
| Desulfotomaculum guttoideum | 3 | 0.01% |
| unclassified Clostridiaceae | 3 | 0.01% |
| Clostridium hylemonae | 3 | 0.01% |
| Ruminococcus gauvreauii | 3 | 0.01% |
| Robinsoniella peoriensis | 3 | 0.01% |
| Parabacteroides merdae | 3 | 0.01% |
| Desulfovibrio fairfieldensis | 3 | 0.01% |
| Helicobacter canadensis | 3 | 0.01% |
| unclassified Ruminococcus | 3 | 0.01% |
| Bacteroides cellulosilyticus | 3 | 0.01% |
| Anaerosporobacter mobilis | 3 | 0.01% |
| Anaerocolumna cellulosilytica | 3 | 0.01% |
| unclassified Paludibacter | 3 | 0.01% |
| Clostridium clariflavum | 3 | 0.01% |
| Clostridium straminisolvens | 3 | 0.01% |
| Clostridium indolis | 3 | 0.01% |
| Hespellia porcina | 3 | 0.01% |
| Prevotella shahii | 3 | 0.01% |
| Coprobacillus cateniformis | 3 | 0.01% |
| Bacteroides caecigallinarum | 3 | 0.01% |
| unclassified Roseburia | 3 | 0.01% |
| Prevotella bryantii | 3 | 0.01% |
| Mycoplasma sualvi | 3 | 0.01% |
| Prevotella scopos | 3 | 0.01% |
| Paeniclostridium sordellii | 3 | 0.01% |
| Prevotella albensis | 3 | 0.01% |
| unclassified Oscillibacter | 3 | 0.01% |
| Lactobacillus amylolyticus | 3 | 0.01% |
| Bifidobacterium choerinum | 3 | 0.01% |
| Clostridium baratii | 3 | 0.01% |
| Ruminococcus champanellensis | 3 | 0.01% |
| Butyrivibrio crossotus | 3 | 0.01% |
| Eubacterium dolichum | 3 | 0.01% |
| Erysipelothrix rhusiopathiae | 3 | 0.01% |
| Lactobacillus oris | 3 | 0.01% |
| Sphaerochaeta coccoides | 3 | 0.01% |
| Bacteroides heparinolyticus | 3 | 0.01% |
| Prevotella melaninogenica | 2 | 0.01% |
| Bacteroides caecicola | 2 | 0.01% |
| unclassified Solobacterium | 2 | 0.01% |
| Hungatella effluvii | 2 | 0.01% |
| Bacteroides salanitronis | 2 | 0.01% |
| Blautia schinkii | 2 | 0.01% |
| Sutterella wadsworthensis | 2 | 0.01% |
| Parasutterella secunda | 2 | 0.01% |
| Anaeromassilibacillus senegalensis | 2 | 0.01% |
| Collinsella intestinalis | 2 | 0.01% |
| Clostridium moniliforme | 2 | 0.01% |
| Lactobacillus agilis | 2 | 0.01% |

| | | |
|---|---|---|
| Allisonella histaminiformans | 2 | 0.01% |
| unclassified Wautersiella | 2 | 0.01% |
| Clostridium aerotolerans | 2 | 0.01% |
| Sphaerochaeta pleomorpha | 2 | 0.01% |
| Acetoanaerobium sticklandii | 2 | 0.01% |
| Clostridium isatidis | 2 | 0.01% |
| Prevotella micans | 2 | 0.01% |
| Acetoanaerobium pronyense | 2 | 0.01% |
| Acetanaerobacterium elongatum | 2 | 0.01% |
| Defluviitalea raffinosedens | 2 | 0.01% |
| Clostridium lactatifermentans | 2 | 0.01% |
| Bulleidia extructa | 2 | 0.01% |
| Parasutterella excrementihominis | 2 | 0.01% |
| unclassified Veillonellaceae | 2 | 0.01% |
| Collinsella stercoris | 2 | 0.01% |
| Bacteroides uniformis | 2 | 0.01% |
| unclassified Lactobacillaceae | 2 | 0.01% |
| Clostridium colicanis | 2 | 0.01% |
| Prevotella amnii | 2 | 0.01% |
| Pseudoflavonifractor capillosus | 2 | 0.01% |
| Holdemania massiliensis | 2 | 0.01% |
| Lactobacillus sakei | 2 | 0.01% |
| unclassified Rikenella | 2 | 0.01% |
| Bifidobacterium longum | 2 | 0.01% |
| Parabacteroides goldsteinii | 2 | 0.01% |
| Prevotella saccharolytica | 2 | 0.01% |
| Tepidibacter mesophilus | 2 | 0.01% |
| Methylocystis echinoides | 2 | 0.01% |
| Desulfovibrio desulfuricans | 2 | 0.01% |
| Porphyromonas pasteri | 2 | 0.01% |
| unclassified Faecalibacterium | 2 | 0.01% |
| Eubacterium ventriosum | 2 | 0.01% |
| Eubacterium siraeum | 2 | 0.01% |
| Lactobacillus antri | 2 | 0.01% |
| Clostridium hungatei | 2 | 0.01% |
| Papillibacter cinnamivorans | 2 | 0.01% |
| Bacteroides gallinaceum | 2 | 0.01% |
| Prevotella marshii | 2 | 0.01% |
| Lactobacillus gasseri | 2 | 0.01% |
| Romboutsia lituseburensis | 2 | 0.01% |
| Eisenbergiella massiliensis | 2 | 0.01% |
| Ethanoligenens harbinense | 2 | 0.01% |
| Caloramator fervidus | 2 | 0.01% |
| Pseudomonas fluorescens | 2 | 0.01% |
| Lactobacillus hominis | 2 | 0.01% |
| Oscillibacter valericigenes | 2 | 0.01% |
| Bacteroides graminisolvens | 2 | 0.01% |
| Lactobacillus psittaci | 2 | 0.01% |
| type II | 2 | 0.01% |
| Casaltella massiliensis | 2 | 0.01% |

| | | |
|---|---|---|
| Eubacterium infirmum | 2 | 0.01% |
| Oceanirhabdus sediminicola | 2 | 0.01% |
| Mogibacterium vescum | 1 | 0% |
| Mucispirillum schaedleri | 1 | 0% |
| Propionispira raffinosivorans | 1 | 0% |
| Natronincola histidinovorans | 1 | 0% |
| unclassified Oscillospira | 1 | 0% |
| beta proteobacterium | 1 | 0% |
| Sutterella parvirubra | 1 | 0% |
| Slackia exigua | 1 | 0% |
| Clostridium sufflavum | 1 | 0% |
| Lactobacillus salivarius | 1 | 0% |
| Blautia hydrogenotrophica | 1 | 0% |
| Clostridium sulfidigenes | 1 | 0% |
| Lachnoanaerobaculum saburreum | 1 | 0% |
| Leptothrix mobilis | 1 | 0% |
| unclassified Holophaga | 1 | 0% |
| Fibrobacter intestinalis | 1 | 0% |
| Lactobacillus coleohominis | 1 | 0% |
| Clostridium amygdalinum | 1 | 0% |
| Helicobacter trogontum | 1 | 0% |
| Bacteroides caccae | 1 | 0% |
| unclassified Actinobacteria | 1 | 0% |
| Alistipes putredinis | 1 | 0% |
| unclassified Subdoligranulum | 1 | 0% |
| unclassified Dialister | 1 | 0% |
| Lactobacillus mucosae | 1 | 0% |
| Terrimicrobium sacchariphilum | 1 | 0% |
| Lactonifactor longoviformis | 1 | 0% |
| unclassified Sutterella | 1 | 0% |
| Pseudobutyrivibrio ruminis | 1 | 0% |
| Bittarella massiliensis | 1 | 0% |
| Selenomonas artemidis | 1 | 0% |
| Acidaminobacter hydrogenoformans | 1 | 0% |
| unclassified Sphingobium | 1 | 0% |
| unclassified Chloracidobacterium | 1 | 0% |
| unclassified Proteus | 1 | 0% |
| Sporosarcina soli | 1 | 0% |
| Prevotella corporis | 1 | 0% |
| Lactobacillus tucceti | 1 | 0% |
| Bacillus gaemokensis | 1 | 0% |
| unclassified Gammaproteobacteria | 1 | 0% |
| Dysgonomonas termitidis | 1 | 0% |
| unclassified Lachnospira | 1 | 0% |
| Shuttleworthia satelles | 1 | 0% |
| Prevotella oulorum | 1 | 0% |
| Campylobacter coli | 1 | 0% |
| unclassified Porphyromonadaceae | 1 | 0% |
| alpha proteobacterium | 1 | 0% |
| unclassified Porphyromonas | 1 | 0% |

| | |
|---|---|
| Thermotalea metallivorans | 1 0% |
| unclassified Longilinea | 1 0% |
| Desulfotomaculum tongense | 1 0% |
| Pedosphaera parvula | 1 0% |
| Conexibacter arvalis | 1 0% |
| Clostridium aminobutyricum | 1 0% |
| Clostridium colinum | 1 0% |
| Caloranaerobacter azorensis | 1 0% |
| unclassified Bacillus | 1 0% |
| unclassified Enterococcus | 1 0% |
| Clostridium perfringens | 1 0% |
| Clostridium bolteae | 1 0% |
| Bacillus niacini | 1 0% |
| Blautia hansenii | 1 0% |
| Pediococcus ethanolidurans | 1 0% |
| Streptococcus azizii | 1 0% |
| Prevotella timonensis | 1 0% |
| Clostridium putrefaciens | 1 0% |
| unclassified Papillibacter | 1 0% |
| Clostridium favososporum | 1 0% |
| Aeriscardovia aeriphila | 1 0% |
| Clostridium longisporum | 1 0% |
| Intestinimonas gabonensis | 1 0% |
| Clostridium scindens | 1 0% |
| Lachnoanaerobaculum umeaense | 1 0% |
| Anaerotruncus colihominis | 1 0% |
| Prevotella oryzae | 1 0% |
| Prevotella fusca | 1 0% |
| Aminicella lysinilytica | 1 0% |
| unclassified Lactobacillus | 1 0% |
| Bacteroides salyersiae | 1 0% |
| Pseudarthrobacter sulfonivorans | 1 0% |
| unclassified Thermomonas | 1 0% |
| Clostridium bornimense | 1 0% |
| Clostridium carnis | 1 0% |
| Georgfuchsia toluolica | 1 0% |
| Prevotella jejuni | 1 0% |
| Clostridium josui | 1 0% |
| Clostridium neopropionicum | 1 0% |
| unclassified Betaproteobacteria | 1 0% |
| Methanosphaera cuniculi | 1 0% |
| Dehalogenimonas alkenigignens | 1 0% |
| Bacteroides neonati | 1 0% |
| Colletotrichum dracaenophilum | 1 0% |
| Prevotella multisaccharivorax | 1 0% |
| unclassified Oribacterium | 1 0% |
| Catabacter hongkongensis | 1 0% |
| Acetatifactor muris | 1 0% |
| Lactobacillus suebicus | 1 0% |
| Desulfosporosinus orientis | 1 0% |

| | | |
|---|---|---|
| Bacillus nealsonii | 1 | 0% |
| unclassified Erysipelotrichia | 1 | 0% |
| Slackia piriformis | 1 | 0% |
| Helicobacter equorum | 1 | 0% |
| Devosia ginsengisoli | 1 | 0% |
| Clostridium glycyrrhizinilyticum | 1 | 0% |
| Lactobacillus gallinarum | 1 | 0% |
| Lactobacillus intestinalis | 1 | 0% |
| Bifidobacterium breve | 1 | 0% |
| Dialister propionicifaciens | 1 | 0% |
| Christensenella minuta | 1 | 0% |
| Gottschalkia acidurici | 1 | 0% |
| Solirubrobacter phytolaccae | 1 | 0% |
| Clostridium methoxybenzovorans | 1 | 0% |
| mixed culture | 1 | 0% |
| Lewinella nigricans | 1 | 0% |
| Bacteroides ovatus | 1 | 0% |
| Roseburia cecicola | 1 | 0% |
| Tepidimicrobium xylanilyticum | 1 | 0% |
| Clostridium taeniosporum | 1 | 0% |
| Sporosarcina psychrophila | 1 | 0% |
| unclassified Olsenella | 1 | 0% |
| Alistipes massiliensis | 1 | 0% |
| unclassified Acidobacterium | 1 | 0% |
| Pediococcus parvulus | 1 | 0% |
| unclassified Erysipelothrix | 1 | 0% |
| Clostridium uliginosum | 1 | 0% |
| Prevotella disiens | 1 | 0% |
| Caloramator proteoclasticus | 1 | 0% |
| Bacteroides paurosaccharolyticus | 1 | 0% |
| Bacillus andreraoultii | 1 | 0% |
| Bacteroides oleiciplenus | 1 | 0% |
| Parvibacter caecicola | 1 | 0% |
| Brevibacillus fulvus | 1 | 0% |
| endosymbiont of | 1 | 0% |
| Salinispora pacifica | 1 | 0% |
| Bacteroides vulgatus | 1 | 0% |
| metal-contaminated soil | 1 | 0% |

**Table S2 Experimental group species after experiment**

| **Experimental group after experiment, species** | **Number of reads** | **Relative abundance** |
|---|---|---|
| Lactobacillus amylovorus | 11170 | 29.45% |
| Prevotella copri | 9558 | 25.2% |
| Megasphaera elsdenii | 1121 | 2.96% |
| Faecalibacterium prausnitzii | 1069 | 2.82% |
| Unclassified | 989 | 2.61% |
| Prevotella stercorea | 877 | 2.31% |
| Collinsella aerofaciens | 841 | 2.22% |
| Gemmiger formicilis | 614 | 1.62% |
| Barnesiella intestinihominis | 570 | 1.5% |
| Alloprevotella rava | 412 | 1.09% |

| | | |
|---|---|---|
| Lactobacillus crispatus | 402 | 1.06% |
| Prevotella oris | 363 | 0.96% |
| Anaerovibrio lipolyticus | 351 | 0.93% |
| Denitrobacterium detoxificans | 347 | 0.91% |
| Enorma massiliensis | 311 | 0.82% |
| Enterorhabdus mucosicola | 304 | 0.8% |
| Prevotella oralis | 304 | 0.8% |
| Lactobacillus panis | 261 | 0.69% |
| Prevotella brevis | 249 | 0.66% |
| Lactobacillus kitasatonis | 231 | 0.61% |
| Olsenella scatoligenes | 226 | 0.6% |
| Roseburia faecis | 215 | 0.57% |
| Oscillospira guilliermondii | 202 | 0.53% |
| Blautia wexlerae | 189 | 0.5% |
| Lactobacillus pontis | 174 | 0.46% |
| Bacteroides caccae | 170 | 0.45% |
| Flintibacter butyricus | 159 | 0.42% |
| Sporobacter termitidis | 155 | 0.41% |
| unclassified Bacteroidales | 155 | 0.41% |
| Escherichia coli | 149 | 0.39% |
| Phascolarctobacterium succinatutens | 148 | 0.39% |
| Eubacterium rectale | 117 | 0.31% |
| Prevotella dentalis | 115 | 0.3% |
| Oscillibacter ruminantium | 112 | 0.3% |
| Lactobacillus delbrueckii | 104 | 0.27% |
| Fusicatenibacter saccharivorans | 102 | 0.27% |
| Butyricicoccus pullicaecorum | 98 | 0.26% |
| Eubacterium ramulus | 86 | 0.23% |
| Paraprevotella clara | 85 | 0.22% |
| Intestinimonas butyriciproducens | 83 | 0.22% |
| Catenibacterium mitsuokai | 81 | 0.21% |
| Bacteroidales oral | 79 | 0.21% |
| Parabacteroides distasonis | 76 | 0.2% |
| Lactobacillus jensenii | 74 | 0.2% |
| Coprococcus catus | 73 | 0.19% |
| Holdemanella biformis | 69 | 0.18% |
| Eubacterium desmolans | 68 | 0.18% |
| Blautia massiliensis | 66 | 0.17% |
| Olsenella uli | 66 | 0.17% |
